# CRISPR/Cas9 Approach to Generate an Auxotrophic BCG Strain for Unmarked Expression of LTAK63 Adjuvant: A Tuberculosis Vaccine Candidate

**DOI:** 10.3389/fimmu.2022.867195

**Published:** 2022-03-30

**Authors:** Luana Moraes, Monalisa Martins Trentini, Dimitrios Fousteris, Silas Fernandes Eto, Ana Marisa Chudzinski-Tavassi, Luciana Cezar de Cerqueira Leite, Alex Issamu Kanno

**Affiliations:** ^1^ Laboratório de Desenvolvimento de Vacinas, Instituto Butantan, São Paulo, Brazil; ^2^ Programa de Pós-Graduação Interunidades em Biotecnologia Universidade de São Paulo - Instituto de Pesquisas Tecnológicas - Instituto Butantan (USP-IPT-IB), São Paulo, Brazil; ^3^ UnivLyon, Université Claude Bernard Lyon 1, Villeurbanne, France; ^4^ Development and Innovation Laboratory, Instituto Butantan, São Paulo, Brazil; ^5^ Center of Excellence in New Target Discovery (CENTD) Special Laboratory, Instituto Butantan, São Paulo, Brazil

**Keywords:** recombinant BCG, CRISPR/Cas9, LTAK63 adjuvant, complemented auxotroph, tuberculosis vaccine

## Abstract

Tuberculosis is one of the deadliest infectious diseases and a huge healthcare burden in many countries. New vaccines, including recombinant BCG-based candidates, are currently under evaluation in clinical trials. Our group previously showed that a recombinant BCG expressing LTAK63 (rBCG-LTAK63), a genetically detoxified subunit A of heat-labile toxin (LT) from *Escherichia coli*, induces improved protection against *Mycobacterium tuberculosis* (*Mtb*) in mouse models. This construct uses a traditional antibiotic resistance marker to enable heterologous expression. In order to avoid the use of these markers, not appropriate for human vaccines, we used CRISPR/Cas9 to generate unmarked mutations in the *lysA* gene, thus obtaining a lysine auxotrophic BCG strain. A mycobacterial vector carrying *lysA* and *ltak63* gene was used to complement the auxotrophic BCG which co-expressed the LTAK63 antigen (rBCGΔ-LTAK63) at comparable levels to the original construct. The intranasal challenge with *Mtb* confirmed the superior protection induced by rBCGΔ-LTAK63 compared to wild-type BCG. Furthermore, mice immunized with rBCGΔ-LTAK63 showed improved lung function. In this work we showed the practical application of CRISPR/Cas9 in the tuberculosis vaccine development field.

## Introduction

The Bacillus Calmette-Guérin (BCG), a live attenuated strain of *Mycobacterium bovis* is the only licensed vaccine against tuberculosis (TB), one of the top 10 causes of mortality (1). BCG is usually administered at birth and is very effective in protecting children against severe forms of the disease. Epidemiological evidence suggests that protection wanes with time and its efficacy in adults against the pulmonary TB is variable (2) contributing to the 10 million new cases and 1.5 million deaths every year (1). Since the development of BCG, a century ago, no new vaccine has been licensed. Therefore, many vaccine candidates are under evaluation as improved vaccines against TB.

Recombinant BCG (rBCG) is an attractive strategy to generate improved TB vaccines. With the development of mycobacterial expression vectors, many strains of rBCG aiming to improve the immune response and protection against TB were generated (3). One of the most advanced vaccine candidates in clinical trials, VPM1002, is based on the rBCG strategy and expresses a pore-forming toxin – cytolysin (4). The expression of toxin derivatives may modulate the immune response and provide improved protection against *M. tuberculosis* (*Mtb*) challenge. We have previously developed a rBCG strain expressing the genetically detoxified subunit A of the heat-labile toxin from *E. coli*, LTAK63, as adjuvant. In comparison to wild-type BCG, immunization of mice with rBCG-LTAK63 increased the Th1 immune response in the lungs (higher IFN-γ, TNF-α, IL-6 and IL-17 production) and long-term immune responses against *Mtb*. rBCG-LTAK63 also conferred improved protection against *Mtb* challenge, including the hypervirulent Beijing strain (5).

The expression of LTAK63 was dependent on a vector containing an antibiotic resistance marker. In order to move forward to clinical trials, it is important to obtain stable antigen expression without antibiotic resistance. Complemented auxotrophic strains are an interesting approach to obtain unmarked expression of heterologous antigens. In this strategy, an essential gene of the biosynthesis of an amino acid is functionally knocked-out. The respective gene is then provided by a complementation plasmid which maintenance is essential for survival and at the same time, the plasmid can be used to co-express antigens of interest (6). Another advantage over antibiotic resistance markers is that the *in vivo* stability of the vector is usually higher (7). We have previously used auxotrophic complementation to obtain an unmarked rBCG strain expressing the genetically detoxified S1 subunit of the pertussis antigen, S1PT, as vaccine against pertussis (8) and bladder cancer immunotherapy (9, 10) as well as presenting increased features of innate immune memory/trained immunity response (11). In that study, the auxotrophic strain was generated using a mycobacteriophage to knock-out the *lysA* gene, involved in the biosynthesis of lysine. This strategy required the insertion of two selection markers to screen for positive mutants and then an additional counter selection step to remove the markers. Even though this process generates an unmarked deletion it stills leaves a chromosomal “scar” (12).

The CRISPR/Cas technology has emerged as a cutting-edge versatile molecular tool for genome manipulation in several organisms. The technique uses a Cas endonuclease (Cas9), a trans-activating RNA (tracrRNA) and a specific targeting sequence (crRNA). This duplex crRNA:tracrRNA (sgRNA) can bind to Cas9 and drives it to the complementary sequence in the genome. The exchange of the crRNA sequence allows targeting of any sequence of interest, with the only condition of being adjacent to a PAM domain (Protospacer Adjacent Motif) (13). So far, applications of CRISPR/Cas in mycobacteria intended to interfere with gene expression (CRISPRi) in order to analyze gene function (14, 15); or to establish the required conditions to generate gene knock-outs in *M. smegmatis*, *M. marinum* and *Mtb* (16).

In this work, we applied CRISPR/Cas9 to generate an auxotrophic BCG, which was complemented to obtain the stable expression of the LTAK63 adjuvant. The immunization with rBCGΔ-LTAK63 showed superior protection against *Mtb* challenge and conferred improved lung function.

## Materials and Methods

### Strains, Media, and Growth Conditions

*Escherichia coli* DH5α, *M. smegmatis* mc^2^ 155, *M. bovis* BCG Danish (American Type Culture Collection, ATCC #35733) and their derivatives were used in the study. *E. coli* was used for the cloning steps and grown in Luria-Bertani (LB) (5 g/L yeast extract, 10 g/L tryptone and 10 g/L NaCl), (Sigma-Aldrich^®^, Merck KGaA, St. Louis, MO, USA). *M. smegmatis* was grown in Middlebrook 7H9 (MB7H9) (Difco, Detroit, MI, USA), supplemented with 0.5% glycerol (Sigma-Aldrich^®^) and 0.05% Tween 80 (Sigma-Aldrich^®^) or plated on Middlebrook 7H10 agar (Difco) supplemented with 0.5% glycerol (MB7H10). BCG was grown in Middlebrook 7H9 supplemented with 10% of OADC (oleic acid-albumin-dextrose-catalase; BBL, Cockeysville, MD, USA), 0.5% glycerol and 0.05% Tween 80 (MB7H9-OADC) or plated on Middlebrook 7H10 agar supplemented with 0.5% glycerol and OADC (MB7H10-OADC). *Mtb* was grown in Middlebrook 7H11 (Difco) supplemented with OADC, glycerol and Tween 80 (MB7H11-OADC). *E. coli* and *M. smegmatis* were grown at 37°C. BCG and *Mtb* were grown at 37°C and 5% CO_2_. When indicated, kanamycin sulphate (20 µg/mL) (Sigma-Aldrich^®^), tetracycline hydrochloride (Tc) (200 ng/mL) (Sigma-Aldrich^®^) and/or L-lysine (40 µg/mL) (Sigma-Aldrich^®^) were added.

### Preparation and Transformation of Competent Cells

Chemically competent *E. coli* DH5α was prepared according to standard protocols (17). Electrocompetent mycobacteria were prepared as previously described (18). For transformation of mycobacteria, 300-500 ng of plasmid DNA were mixed with competent cells and electroporation performed using a Gene Pulser II device (BioRad, Hercules, CA, UK). Cells were recovered in MB7H9 and plated onto MB7H10 agar until the appearance of visible colonies. Kanamycin, tetracycline, or/and lysine were added to the media, as described above, when required.

### Construction of the Mycobacterial CRISPR/Cas9 All-In-One Vectors

**Figure 1:**
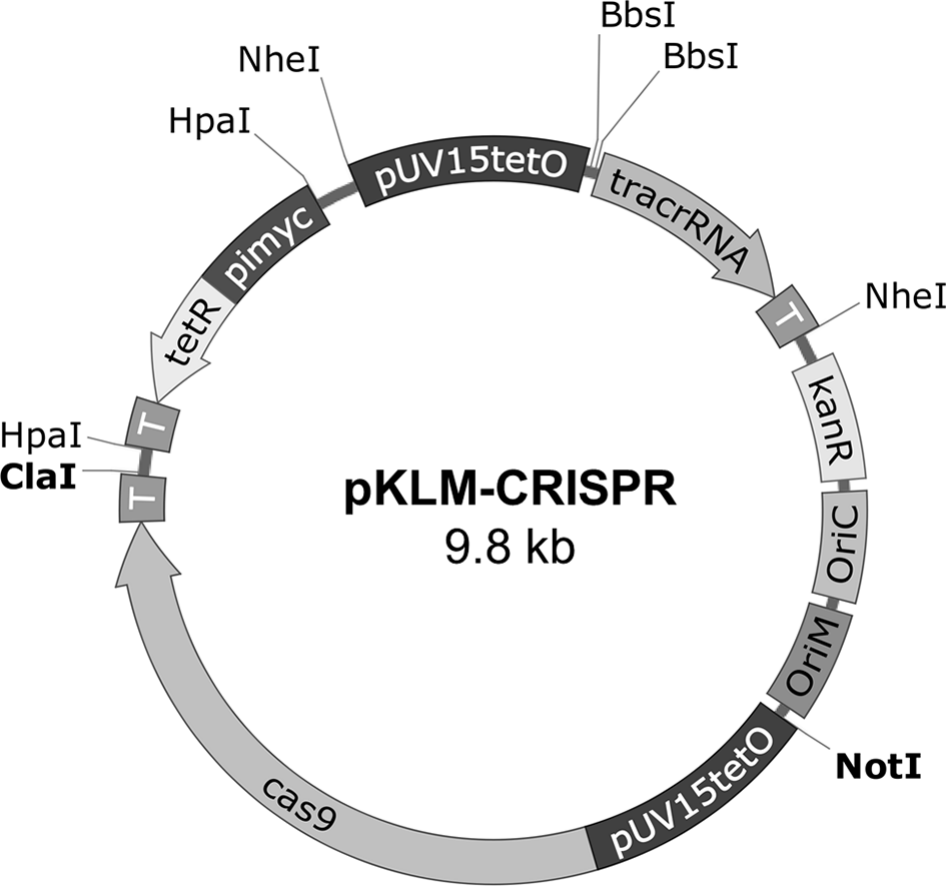
Schematic representation of the pKLM-CRISPR vector. The pKLM-CRISPR vector, contains the expression cassette for codon optimized *cas9* gene and a cassette for expression of sgRNA, both under regulation of pUV15tetO, a tetracycline-inducible promoter. The repressor tetR is expressed constitutively by the pimyc promoter. Transcriptional terminators (T) were introduced after each expression cassette. The restriction sites used for cloning are also indicated. The two BbsI sites were introduced to facilitate the cloning of the crRNA sequences upstream of tracrRNA. The vectors also contain a kanamycin resistance marker (KanR); origin of replication in *E. coli* (oriC) and mycobacteria (oriM).

Codon-optimized *cas9* expression cassette, the Tet regulator (*tetR*) cassette and the tracrRNA sequences (Genscript, Piscataway, NJ, USA) were sequentially cloned into pJH152 (a kind gift from Dr. Stewart Cole, EPFL, Lausanne, France) using the restriction enzymes NotI/ClaI, HpaI and NheI (New England Biolabs, Ipswich, MA, USA), respectively, originating pKLM-CRISPR (**Figure 1**). Selection of crRNA was made using the Cas-Designer tool at R-GENOME website (http://www.rgenome.net/cas-designer/). Each crRNAs sequence was inserted using BbsI (New England Biolabs) upstream of tracrRNA in pKLM-CRISPR, generating the sgRNA sequences. The expression of Cas9 and sgRNA are both controlled by tetracycline-inducible promoters (pUV15tetO). The T4g32 transcriptional terminator was added to each cassette end (T). Orientation of the cloned fragments according to **Figure 1** were confirmed by Sanger sequencing. Vectors including crRNA aiming to knock-out *lysA* were named pKLM-CRISPR-*lysA(x)*, where *(x)* represents different crRNA sequences. The list of plasmids and oligonucleotides used are shown in **Supplementary Tables S1, S2**. Alignment of *lysA* from BCG and *M. smegmatis* as well as the relative position of sgRNA targets are represented in **Supplementary Figure 1**.

### Construction of Complementation Vectors Expressing LysA and LTAK63

**Figure 2:**
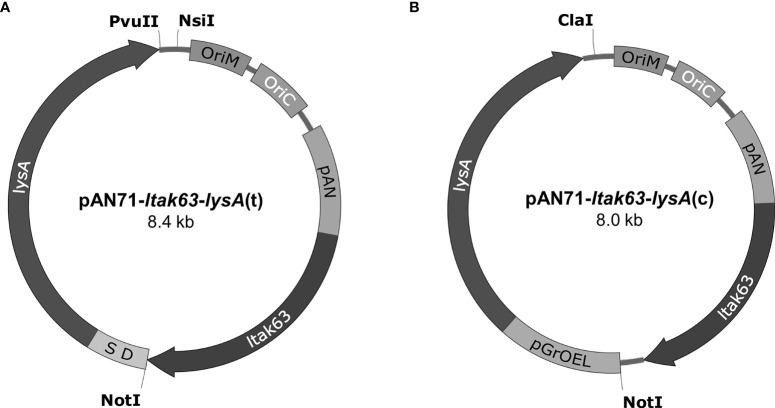
Vectors pAN71-*ltak63-lysA*(t) and pAN71-*ltak63-lysA*(c) used for the complementation of lysine auxotrophic strains. **(A)** In pAN71-*ltak63-lysA*(t) the *lysA* gene is in tandem with *ltak63*, including a Shine-Dalgarno sequence (SD) between the genes. **(B)** In pAN71-*ltak63-lysA*(c) the *lysA* gene is under the control of the pGrOEL promoter. The KanR site was truncated by restriction digestion with NsiI or ClaI, respectively and self- ligated. Both plasmids share the same features such as oriM and oriC, the pAN promoter and the *ltak63* sequence.

The pAN71-*ltak63* vector containing pAN promoter and driving low expression of the LTAK63 adjuvant was previously constructed by our group (5). A PCR-amplified *lysA* gene (containing a Shine-Dalgarno, SD), or a *lysA* expression cassette digested from pJH152 were used. The pAN71-*ltak63* vector was digested with NotI/PvuII (New England Biolabs) and the *lysA* fragment inserted in tandem with *ltak63* under the same pAN promoter, generating pAN71-*ltak63-lysA*(t) (**Figure 2A**). In this construct, the kanamycin resistance marker was removed by digestion with NsiI (New England Biolabs) and self-ligated. The pAN71-*ltak63* vector was also digested with NotI/ClaI (New England Biolabs) to clone the *lysA* expression cassette, generating pAN71-*ltak63-lysA*(c) (**Figure 2B**). The kanamycin resistance marker was removed by digestion with ClaI (New England Biolabs) and self-ligated. Both vectors were electroporated into *M. smegmatis* mc^2^ 1493 (lysine auxotroph) (19) and transformants plated onto MB7H10. Transformants were then grown in MB7H9 with and without kanamycin to confirm construction of the unmarked pAN71-*ltak63-lysA* vectors. Plasmid extraction from *M. smegmatis* was performed using Wizard Plus SV Minipreps DNA Purification kit (Promega, Madison, WI, USA) and the extracted plasmids were used to transform the lysine auxotrophic BCG (BCGΔ*lysA*).

### Induction of Cas9 Expression in Mycobacteria

After transformation of *M. smegmatis* and BCG with pKLM-CRISPR-*lysA(x)*, kanamycin-resistant colonies were recovered and cultured in 5 mL of MB7H9 and MB7H9-OADC, respectively. These cultures were used as pre-inoculum in a fresh culture starting at OD_600_ 0.1. After 2 h (*M. smegmatis*) or 24 h (BCG) of incubation, tetracycline was added to induce expression of Cas9, and the culture was maintained at 37°C for 4-24 h (*M. smegmatis*) or 24-120 h (BCG). After the induction, bacteria were lysed by sonication using an ultrasonic processor GE100 (GE Healthcare, Chicago, IL, USA) and protein extracts separated by SDS-PAGE (BioRad, Hercules, CA, UK), transferred to PVDF membranes (GE Healthcare) and blocked with 5% non-fat dry milk at 4°C for 16 h. The membrane was probed using monoclonal anti-Cas9 antibodies (7A9-3A3, 1:1,000) (Santa Cruz Biotechnology, Dallas, TX, USA) incubated for 90 min and anti-mouse IgG conjugated with peroxidase (A6782, 1:1,000) (Sigma-Aldrich^®^) for 60 min. *E. coli* DH5α transformed with pCas (20) was used as positive control for Cas9 expression. Chemiluminescent signal was developed using ECL Prime Western Blotting System (GE Healthcare) and images acquired with the LAS4000 digital imaging system (GE Healthcare).

### Screening and Characterization of Knock-Out Mutants

After tetracycline induction the cultures were plated onto MB7H10 or MB7H10-OADC, both supplemented with lysine and kanamycin and incubated at 37°C until the appearance of visible colonies. Single colonies were then transferred to mirror plates either containing or not lysine, both in presence of kanamycin. Growth only in the lysine-containing plate revealed positive knock-out clones (Lysine-KO). The Lysine-KO clones were used as template for PCR amplification of the *lysA* region and sequencing. The *lysA* sequences from the Lysine-KO clones were compared with the reference sequences in UNIPROT database (*M. smegmatis*-Q9X5M1 and BCG-P9WIU7).

### *In Vitro* Stability of BCG Functional KO

To assess reversion to the wild-type phenotype during *in vitro* culturing, rBCGΔ*lysA* strains were serially passaged weekly for up to 8 passages when the cells were plated on MB7H10-OADC containing or not lysine. Additionally, the curing of pKLM-CRISPR-*lysA(x)* was assessed by plating rBCGΔ*lysA* onto mirror plates containing or not kanamycin. Growth only in the plate lacking kanamycin indicated plasmid loss.

### Complementation of Auxotrophic Strains

Competent BCGΔ*lysA* were prepared as previously described and transformed with pAN71-*ltak63-lysA*(t) and pAN71-*ltak63-lysA*(c), generating rBCGΔ-LTAK63(t or c), respectively. Selected clones were grown in MB7H9-OADC until an OD_600_ 1.0, when bacteria were recovered, and protein extracts used to detect the expression of LTAK63 by Western blot (20). Detection of LTAK63 was performed using anti-serum of mice previously immunized with rLTK63 (1:1,000) incubated for 60 min and an anti-mouse IgG antibody conjugated with peroxidase (A6782, 1:3,000 Sigma-Aldrich^®^) incubated for 60 min. Additionally, growth curves of complemented auxotrophs were compared to wild-type BCG to determine whether rBCGΔ-LTAK63(t or c) would show altered *in vitro* growth.

### Animals and Immunization

All animal experiments were performed according to Brazilian and international guidelines on animal experimentation and approved by the Ethics Committee of Instituto Butantan, São Paulo-SP (CEUAIB), (Permit number 8591010817). Five to eight-week-old female BALB/c mice were obtained from the Central Animal Facility of the Instituto Butantan, SP, Brazil. BALB/c were immunized through the subcutaneous route (s.c.) with a single dose of BCG or rBCGΔ-LTAK63(t) (1x10^6^ CFU/100 µL), or saline and challenged after 90 days *via* the intranasal route (i.n.) with *M. tuberculosis* H37Rv (500 CFU/50 µL). Thirty days after challenge, the lungs were collected, homogenized, and 20 µL of serial dilutions (10^-1^, 10^-2^ and 10^-3^) were plated on MB7H11-OADC at 37°C and 5% CO_2_ for CFU counting.

### Histopathology and Quantification of the Lung Inflammation

Thirty days after *Mtb* challenge, lung tissue samples were collected, preserved in 10% neutral buffered formalin, embedded in paraffin, cut into 5-6 μm sections, and stained with hematoxylin and eosin (H&E). The severity of inflammation in the mouse lungs was assessed according to (21). In summary, H&E-stained lung sections were photographed at 40 x magnification using a microscope (Nikon Eclipse Ti-S) coupled to a digital camera (DS-Fi1c, Nikon). Image analysis software (ImageJ, National Institutes of Health, USA) was used to determine the pulmonary area affected. The inflammatory area was measured according to (21) and the functional lung area is represented by the intra-alveolar regions in the lung tissue determined using morphometric analysis according to (22). Briefly, five images at 40 x magnification per lung lobule, totaling 25 images per treatment, were randomly selected, and analyzed for the qualitative evaluation of the cell infiltrate and intra-alveolar regions. To measure the areas of interest the images were transformed into 8-bit and treated with threshold and percentage of the measured area. For leukocyte counting, the Color Deconvolution 2 plugin were used to visualize and separate nuclei from the cytoplasm. For cell counting, the Cell Counter plugin was used. This analysis is used to facilitate the differential counting of segmented and mononuclear nuclei.

### Statistical Analysis

Statistical analysis was performed using the GraphPad Prism (GraphPad Software, Inc.). The significance of differences among groups was calculated by unpaired parametric two-way Student**’**s t-test as described in the figure legends. Differences between mean values were considered significant when p < 0.05.

## Results

### Inducible Expression of Cas9 in Mycobacteria

**Figure 3:**
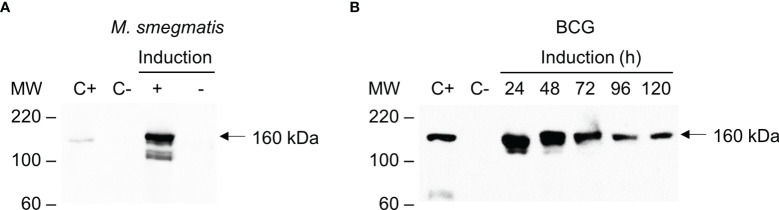
Inducible expression of Cas9 in *M. smegmatis* and BCG. *E. coli* transformed with pCas9 was used as positive control (C+). Total protein extracts of wild-type mycobacterial strains were used as negative controls (C-). **(A)** Western blot of total protein extracts of *M. smegmatis* transformed with pKLM-CRISPR vector with either Cas9 expression induced (+) or not induced (-) with tetracycline. **(B)** Western blot of the expression of Cas9 in BCG induced with tetracycline over time (24, 48, 72, 96 or 120 h). The Western blots were probed with monoclonal anti-Cas9 antibody (1:1,000). Molecular weight markers (MW) are indicated in the left and the expected molecular weight of Cas9 indicated by an arrow (~160 kDa).

Transformation of *M. smegmatis* and BCG with pKLM-CRISPR showed that both were able to express Cas9, as determined by the Western blot (~160 kDa). In *M. smegmatis*, the highest expression of Cas9 was observed at 8 h after the induction in a concentration of 200 ng/mL of tetracycline (**Supplementary Figure 2** and **Figure 3A**). In BCG, the highest expression of Cas9 was between 24-48 h after the induction, with decreasing levels being observed from that time until 120 h (**Figure 3B**). Attempts to express the wild-type *S. pyogenes* Cas9 in mycobacteria were unsuccessful (data not shown).

### Phenotypic Screening and Characterization of SmegΔ*lysA* and BCGΔ*lysA*

**Figure 4:**
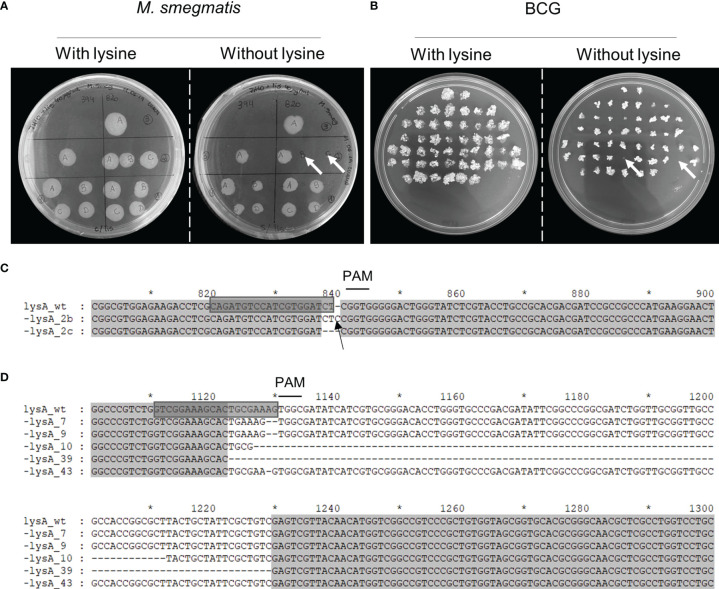
Phenotypic screening and genotypic characterization of SmegΔ*lysA* and BCGΔ*lysA*. After the induction of Cas9, the cultures were seeded on lysine-supplemented plates to recover all viable bacteria. The colonies of **(A)** *M. smegmatis*, and **(B)** BCG were then seeded on mirror plates with and without lysine. The colonies that did not grow on plates without lysine (white arrows) indicate a positive knock-out. The *lysA* genes from **(C)** SmegΔ*lysA*, and **(D)** BCGΔ*lysA* were sequenced and compared to the wild-type sequence. The sgRNA used to target *lysA* is highlighted (grey box); the deleted nucleotides are represented by the dashed line; the inserted nucleotide is pointed out with a black arrow and the PAM site (NGG) is indicated with a line above. Numbering and asterisks represent the nucleotide positions regarding the full *lysA* gene sequence.

Mycobacteria were transformed with the gene editing vectors (pKLM-CRISPR containing the specific crRNAs to target *lysA* (n=3 for *M. smegmatis* and n=2 for BCG) (**Supplementary Table 2**), and the expression of Cas9 was induced by tetracycline. After induction, the culture was plated on solid media containing kanamycin to obtain isolated colonies. These were transferred to mirror plates with and without lysine to detect the functional knock-out of LysA. The transformation of *M. smegmatis* with the vector containing crRNA-*lysA820*-Smeg, resulted in 2 out of 8 colonies (25%) that did not grow in the absence of lysine (SmegΔ*lysA*) (**Figure 4A**). The induction with crRNA-*lysA394*-Smeg did not generate any functional knock-out (0/5), while crRNA-*lysA123*-Smeg did not produce any transformant. In BCG transformed with the vectors containing crRNA-*lysA*88-BCG, 2 out of 50 colonies (4%) did not grow without the supplementation of lysine (BCGΔ*lysA*) (**Figure 4B**). No functional knock-outs were obtained using the vector containing crRNA-*lysA20*-BCG. In the genotypic analysis of *M. smegmatis*, we observed the addition of a single nucleotide (*lysA*_2b) and the deletion of two nucleotides (*lysA*_2c) next to the PAM site (**Figure 4C**). In BCG, we performed an additional round of induction (independent experiment), obtaining a total of five functional knock-outs. Sequencing analysis of BCGΔ*lysA* demonstrated the deletion of two nucleotides (*lysA*_7 and *lysA*_9), deletion of larger fragments (*lysA*_10 and *lysA*_39), 83 and 107 bp (**Figure 4D** and **Supplementary Figure 3**), and a single nucleotide deletion (*lysA*_43). The *in-silico* translation of these sequences revealed that the mutations results in the early interruption of translation or frameshifts impairing the correct translation of LysA (**Supplementary Figure 4**). The possibility of reversion from the auxotrophic phenotype was evaluated by subculturing BCGΔ*lysA* and observation of growth without supplementation of lysine. Even after 8 passages, without the supplementation of lysine, no prototrophic colony was observed (**Supplementary Figure 5**).

### LTAK63 Adjuvant Expression in Auxotrophic Mycobacteria

**Figure 5:**
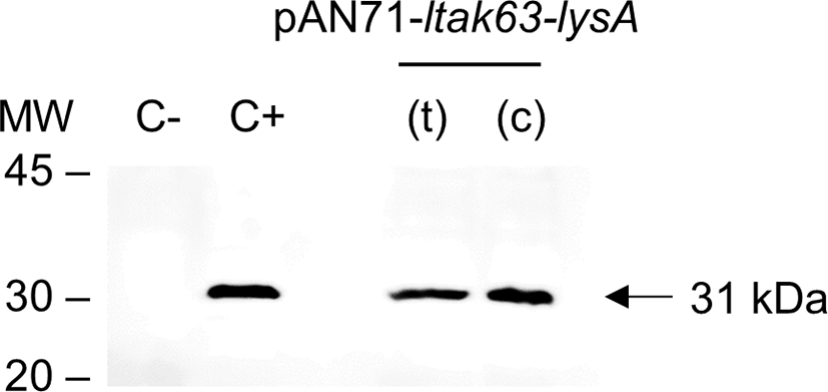
Expression of LTAK63 in complemented auxotrophic BCG. Western blot of total protein extracts of wild-type BCG (C-); rBCG-LTAK63 (C+) and complemented auxotrophic strains obtained by transformation of BCG*ΔlysA* with pAN71-*ltak63-lysA* either in the tandem construct (t) or cassette (c). Western blots were probed with mouse anti-serum raised against rLTAK63 (1:1,000). Molecular weight markers (MW) are indicated and the expected molecular weight of LTAK63 is indicated by an arrow (~31 kDa).

Once reversion to the wild-type phenotype was excluded, the BCG*ΔlysA* lysA_39 (mutant containing the deletion of 107 bp) was selected to be complemented. Before the complementation, the pKLM-CRISPR plasmid was cured. In a single incubation without kanamycin, 35% of the colonies showed plasmid loss (**Supplementary Figure 6**). A single colony was selected and prepared as competent cells. The BCG*ΔlysA* was then transformed with the complementation vectors, pAN71-*ltak63-lysA*(t) and pAN71-*ltak63-lysA*(c) and protein extracts used to detect the expression of LTAK63 (**Figure 5**). More importantly, either when driven by the vectors with *lysA* in tandem or as an expression cassette, the level of LTAK63 expression was comparable to that observed in BCG transformed with the pAN71-*ltak63* vector.

### *In Vitro* Growth of Complemented Auxotrophic BCG

We investigated whether the complemented auxotrophic BCG would show distinct *in vitro* growth in comparison to wild-type mycobacteria. It can be observed that both recombinant BCG strains showed growth comparable to the wild-type (**Supplementary Figure 7**). The rBCGΔ-LTAK63(t) was selected for the *in vivo* experiments, hereafter, named rBCGΔ-LTAK63.

### Protection of Immunized Mice Against Intranasal Challenge With *Mtb*

**Figure 6:**
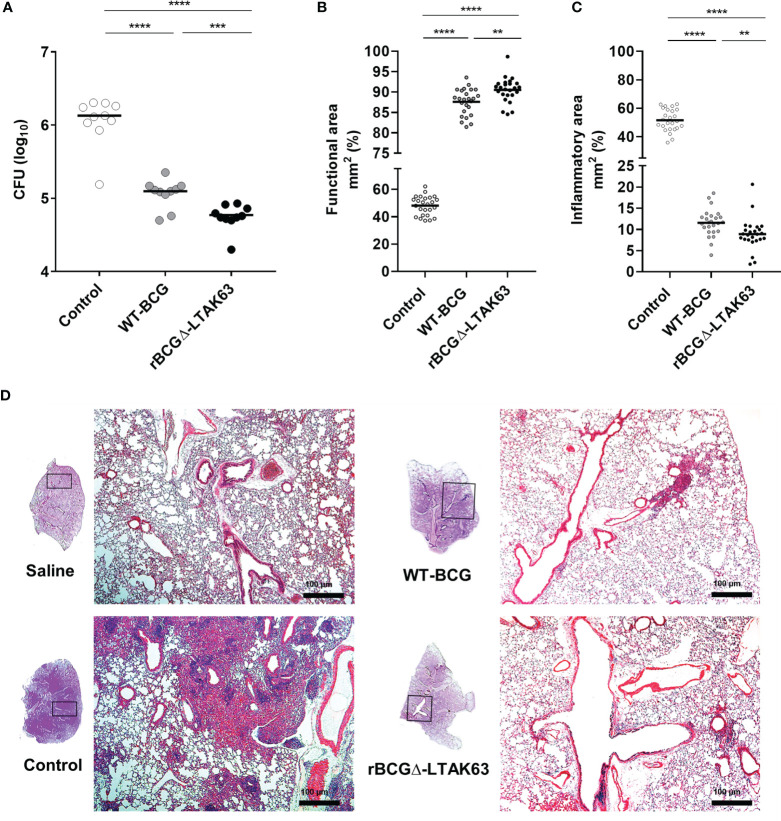
Protection against *Mtb* challenge induced by immunization. Groups of BALB/c mice (n=10/group) immunized s.c. with BCG (WT-BCG), rBCGΔ-LTAK63, or not immunized (Control) were challenged i.n. with 500 CFU of *Mtb*. Thirty days after challenge, the lung was recovered to evaluate CFU **(A)**, functional lung area, represented by the intra-alveolar space **(B)** and inflammatory area **(C)**, represented by the inflammatory infiltrate of lung sections stained with H&E. Functional area and lung inflammation scores are presented as the mean percentage of inflammation for each mouse and the infiltrate is presented as cell counts per mm². **p < 0.01, ***p < 0.001 and ****p < 0.0001. Representative histopathology of lungs from naïve mice (Saline), infected only (Control), or immunized and challenged (BCG and rBCGΔ-LTAK63). Lung sections were stained with H&E (bar, 100 µm) **(D)**. Challenge experiments were performed twice.

**Figure 7:**
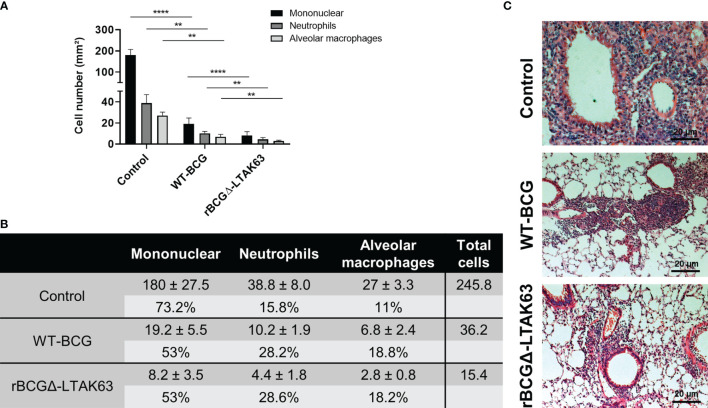
Characterization of the cellular infiltrate in the lungs. Total number of mononuclear cells, neutrophils, and alveolar macrophages in the perivascular and peribronchiolar infiltrates in the lungs of BALB/c mice infected only (Control), or immunized and challenged (BCG and rBCGΔ-LTAK63) represented as cell counts per mm². **p < 0.01 and ****p < 0.0001 **(A)** and the actual cell numbers, percentage and total cell count in each group **(B)**. Five images at 40 x magnification per lung lobule, totaling 25 images per treatment, were randomly selected. For cell counting, ImageJ software and the Color Deconvolution 2 plugin were used to visualize and separate nuclei from the cytoplasm. Representative histopathology of lungs sections stained with H&E (bar, 20 µm) **(C)**. Challenge experiments were performed twice.

BALB/c mice were immunized with BCG, rBCGΔ-LTAK63 or not immunized and were challenged with *Mtb* 90 days after. The group immunized with rBCGΔ-LTAK63 showed a 1.5 log CFU reduction in the lungs when compared to the control group and 0.5 log reduction when compared to the BCG group (**Figure 6A**). Histopathological findings typical in a *Mtb* infection in the mouse model showed a granulomatous inflammatory process spread across the infected lungs. Immunization with rBCGΔ-LTAK63 showed increased protection of the lung tissue compared to BCG as evaluated by the functional lung area (**Figure 6B**). The effect of rBCGΔ-LTAK63 on leukocyte migration differs from that of BCG, displaying a reduction in the inflammatory score (**Figure 6C**). The improvement of the histopathological lesions is shown in the representative lung sections. Mice immunized with rBCGΔ-LTAK63 showed fewer lesions consisting of perivascular and peribronchiolar inflammatory infiltrates when compared with BCG-immunized mice (**Figure 6D**). Characterization of the cellular infiltrate showed the presence of mononuclear cells, neutrophils, and alveolar macrophages in the perivascular and peribronchiolar infiltrates; a general decrease in the total number of these cell types in the lungs of the groups immunized with BCG or rBCGΔ-LTAK63 indicates improved protection against lung pathology upon vaccination (**Figure 7**).

## Discussion

BCG is the only licensed vaccine against tuberculosis. It is especially important for children to prevent the development of severe forms of TB. However, its efficacy wanes over time and adults are less protected. In order to develop improved vaccines against tuberculosis, thousands of potential candidates are in the discovery phase, hundreds have undergone preclinical trials in animal models, but only few candidates passed to clinical studies in humans (23). Among the most promising vaccine candidates are the live attenuated VPM1002 and MTBVAC. Interestingly, both vaccines required the generation of genomic mutations. While in VPM1002 it was necessary to disrupt the *ureC* gene – to provide an optimal environment for the cytolysin’s activity. The insertion of lysteriolysin gene at the *ureC* locus demanded the use of a hygromycin marker for selection, which removal “was technically extremely challenging” (24). On the other hand, MTBVAC was obtained by deletion of two independent genes, *fadD26* and *phoP* (25). To achieve such mutations, a stepwise insertion/deletion process, comprising 4 different steps, was necessary (26). All these time-consuming and labor-intensive procedures could be avoided with the use of CRISPR/Cas9.

In our approach, we constructed an all-in-one vector containing the CRISPR/Cas9 elements required for the generation of the gene knock-outs in one step. To enable better control over the expression of Cas9 and sgRNA, we chose to use tetracycline-inducible promoters for both. The peak of Cas9 expression was observed at 8 h in *M. smegmatis* and 24-48 h for BCG. Lower levels of Cas9 expression were observed with prolonged incubation. Other studies have also observed that longer incubations are not necessary to induce Cas9 or other nucleases (13). The use and characterization of inducible promoters in CRISPR/Cas systems are important since continuous induction could lead to undesired mutations at unknown genomic *loci*.

To obtain the disruption of *lysA*, three sgRNA for *M. smegmatis* and two for BCG were designed. In *M. smegmatis*, the transformation with one of the selected sgRNA constructs did not produce any transformants. Between the two that did generate transformants, functional knock-outs were observed in only one. In BCG, the two constructs produced transformants, but knock-outs were observed in only one. Interestingly, plasmid constructs that induced the functional knock-out of *lysA* contained sgRNAs targeting the positive strand. It should be noted that we used a phenotypic screening to evaluate knock-outs and therefore silent mutations e.g. those maintaining the original reading frame, were not detected by this approach. All the mutations induced by CRISPR/Cas9 were characterized by the removal of nucleotides which indicates the action of non-homologous end-joining (NHEJ) DNA repair mechanisms in these cases. In *M. smegmatis*, one of the knock-outs had an extra nucleotide near the PAM site which is also a possibility in this particular strain (16). While most bacteria employ homologous recombination (HR) to repair double-strand breaks (DBS) in their DNA, mycobacteria have developed additional repair mechanisms. Besides HR, NHEJ and SSA (single-strand annealing) are functional and described as not redundant but rather defined as distinct DSB repair pathways (27). As CRISPR/Cas9 exploits the DNA repair mechanisms to generate the mutations in the host genomes, further studies to understand the interplay between these repair systems are necessary. For instance, it may be possible to favor the HR repair and the consequently knock-in of sequences by disrupting key gene proteins (such as *ku* and *ligD*) to abolish NHEJ-mediated repair, or by inducing the overexpression of HR-related proteins (27).

We applied CRISPR/Cas9 to obtain an unmarked and scarless gene editing, thus resulting in a lysine auxotrophic BCG strain which was later complemented to stably express the LTAK63 adjuvant. The use of auxotrophic complementation is an interesting approach to obtain unmarked heterologous expression and increase the stability of the construct. On the other hand, it can also result in impaired growth since the level of LysA expressed can be different from that of the original wild-type strain. Here, we evaluated two different constructs using *lysA* complementation either in tandem with *ltak63* [pAN71-*ltak63-lysA*(t)] or each gene with its own expression cassette [pAN71-*ltak63-lysA*(c)]. The construct in tandem is driven by the pAN promoter, which is considered a weak mycobacterial promoter (28). If the expression of LysA is too low, then it could also affect BCG’s fitness and the protection induced against *Mtb*. Alternatively, the pAN71-*ltak63-lysA*(c) construct includes another promoter for the expression of *lysA*, the pGrOEL. The addition of another expression cassette may decrease plasmid stability and compromise the expression of LTAK63. Our data show that whichever strategy used for the expression of LTAK63 and LysA, the growth of recombinant BCG strains is comparable to the wild-type strain. More importantly, the complemented strains generated here were able to express the LTAK63 antigen at levels comparable to the original rBCG-LTAK63 construct, which is imperative in order to obtain improved protection against *Mtb* (5).

Immunization of mice with the complemented auxotrophic BCG expressing LTAK63 (rBCGΔ-LTAK63) confirmed its superior protection against *Mtb* challenge. Previews reports demonstrated that mice immunized with rBCG-LTAK63 and challenged intratracheally with *Mtb* displayed a 1-2 log reduction of CFU in the lungs in comparison to wild-type BCG. Here, we observed that mice immunized with rBCGΔ-LTAK63 and challenged with *Mtb* exhibit a 0.5 log reduction in comparison to wild-type BCG. This difference may be explained by the distinct routes and bacterial loads involved in the challenge (intratracheal, 1x10^5^ CFU, and intranasal, 500 CFU) (5). The intranasal route has the advantage of being less invasive and better mimics the natural *Mtb* infection (29). Accordingly, the protective effects on lung tissue during the clinical course of the infection was clear in the groups of mice immunized with BCG or rBCGΔ-LTAK63. However, it is notorious that immunization with rBCGΔ-LTAK63 intensified the protective effects evaluated as functional lung area and modulated the leukocyte response in comparison to BCG. The reduction in the cellular infiltrate after the *Mtb* challenge may, in fact, be associated with a faster and improved resolution of the infection induced by the immunization of rBCGΔ-LTAK63.

In this work we produced lysine-deficient mutants using a one-step induction of CRISPR/Cas9; furthermore, we demonstrated the efficient complementation with *lysA*-containing vectors also expressing LTAK63 adjuvant. Immunization with rBCGΔ-LTAK63 induced better protection against *Mtb* challenge. The data presented here shows the practical application of CRISPR/Cas9 towards the generation of new and improved TB vaccines.

## Data Availability Statement

The original contributions presented in the study are included in the article/**Supplementary Material**. Further inquiries can be directed to the corresponding author.

## Ethics Statement

The animal study was reviewed and approved by Ethics Committee of Instituto Butantan (CEUAIB) (Permit number 8591010817).

## Author Contributions

LL and AK conceived and designed the experiments. LM, MT, DF, and SE performed the experiments and collected data. LM, MT, DF, SE, AC-T, LL, and AK processed and analyzed the data. LM, MT, SE, LL, and AK wrote the manuscript. All authors contributed to the article and approved the submitted version.

## Funding

We acknowledge the support from FAPESP (Projects 2017/24832-6, 2017/17218-0 and 2019/06454-0) and Fundação Butantan.

## Conflict of Interest

LL has a patent application on the use of rBCG-LTAK63 as vaccine against *Mtb*. The funders had no role in the design of the study, in the collection, analyses, or interpretation of data, in the writing of the manuscript, or in the decision to publish the results.

The remaining authors declare that the research was conducted in the absence of any commercial or financial relationships that could be construed as a potential conflict of interest.

## Publisher’s Note

All claims expressed in this article are solely those of the authors and do not necessarily represent those of their affiliated organizations, or those of the publisher, the editors and the reviewers. Any product that may be evaluated in this article, or claim that may be made by its manufacturer, is not guaranteed or endorsed by the publisher.
