# Supplementary material for: CRISPR/Cas9 Approach to Generate an Auxotrophic BCG Strain for Unmarked Expression of LTAK63 Adjuvant: A Tuberculosis Vaccine Candidate
Source: Front Immunol. 2022 Mar 30;13:867195. doi: 10.3389/fimmu.2022.867195 (PMC9005855; doi:10.3389/fimmu.2022.867195)
Supplement: Supplementary file 1 [file DataSheet_1.docx]

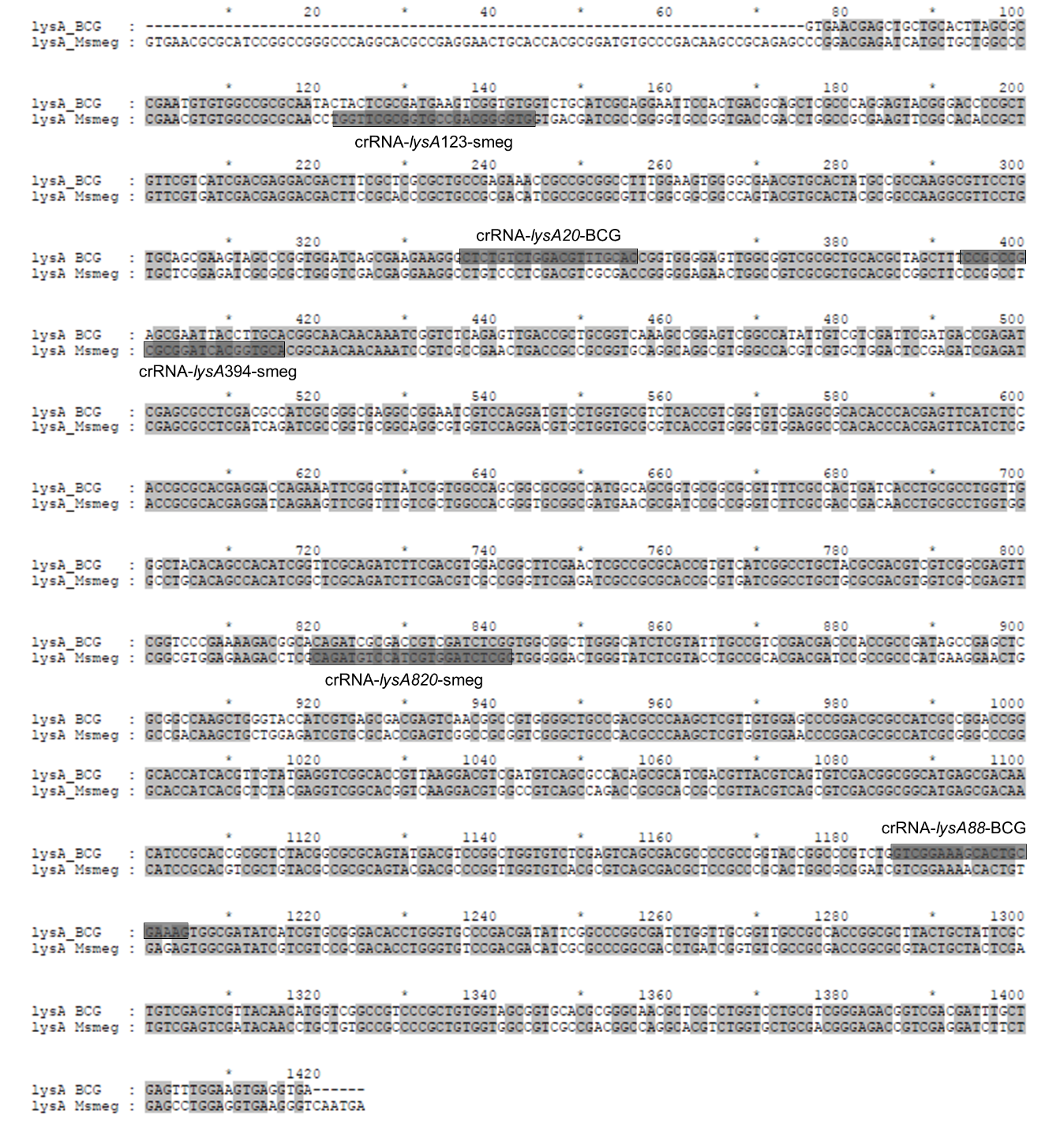


**Supplementary Figure 1. Alignment of BCG and *M. smegmatis* lysA gene sequences**. The regions of similarity between the sequences are highlighted in grey. The crRNAs used to target the *lysA* gene in BCG and *M smegmatis* are marked in their positions with their respective names.


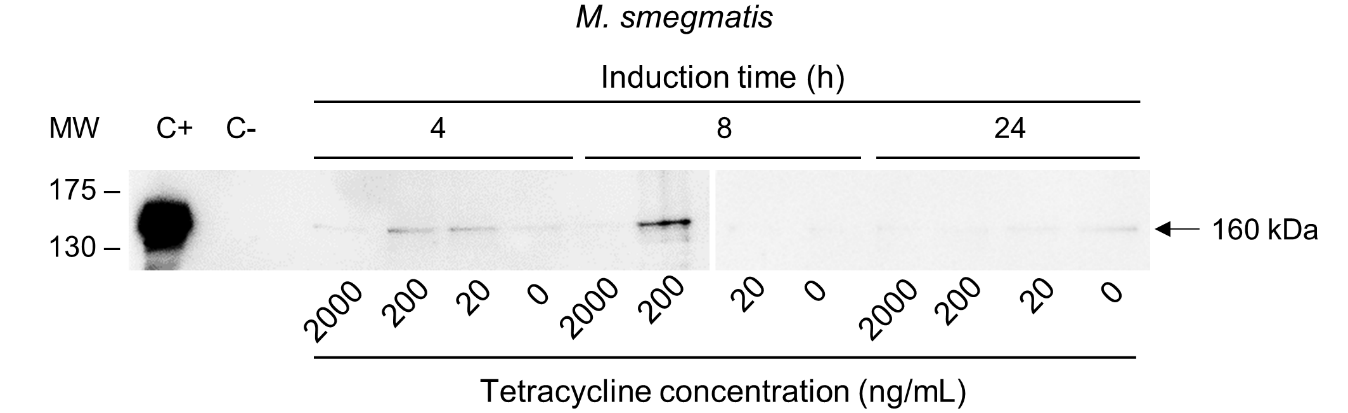


**Supplementary Figure 2. Different levels of Cas9 expression depending on tetracycline concentration and time of induction.** Different concentrations of tetracycline were evaluated (2000, 200, 20, 0 ng**/**mL) in different times of induction for *M. smegmatis* (4, 8, 24 h). Molecular Weight (MW); *E. coli* transformed with pCas9 (C+); Total extract of wild-type *M. smegmatis* (C-). The expected size of Cas9 (160 kDa) indicated by an arrow.


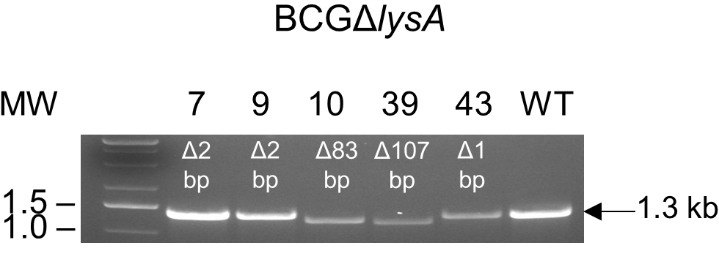


**Supplementary Figure 3. Differences in band sizes observed by PCR after genome editing.** The *lysA* gene sequence from five different phenotypically confirmed BCGΔ*lysA* colonies (7, 9, 10, 39, and 43) was PCR-amplified using specific primers (see Supplementary Table 2). The two BCGΔ*lysA* strains with the deletions of 83 and 107 bp (strains 10 and 39, respectively) exhibited a different and visible pattern of electrophoresis observed on a 1% agarose gel.


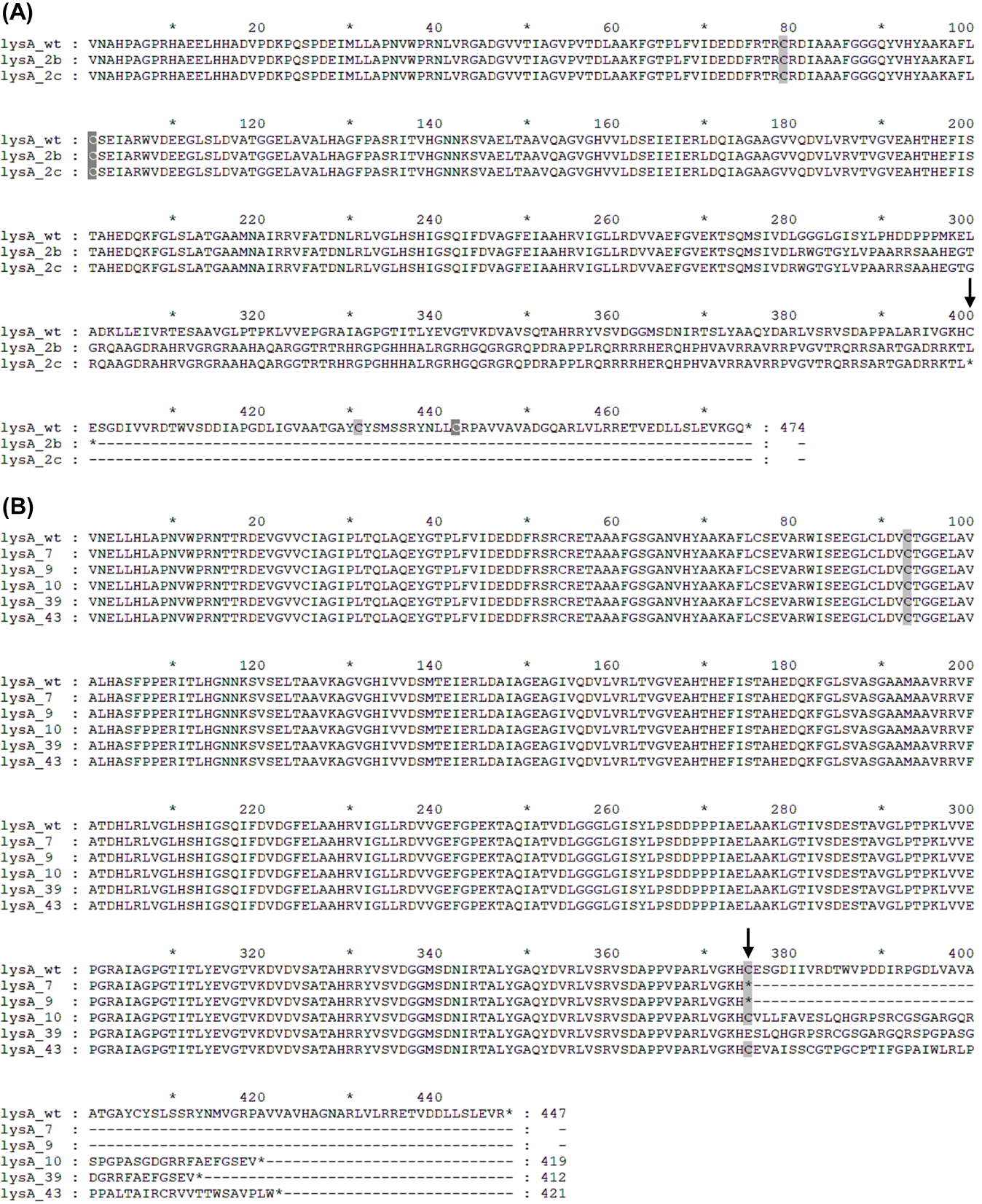


**Supplementary Figure 4. Mutations in BCGΔ*lysA* results in a truncated protein sequence.** The PCR-amplified fragment of *lysA* gene from all SmegΔ*lysA* (A) and BCGΔ*lysA* (B) strains and the native BCG and *M. smegmatis* was sequenced by Sanger sequencing. Translated sequences were aligned on ClustalX2 and compared to the original unmutated *lysA* aminoacid sequence. STOP codon is represented by an asterisk. Gray shading shows the cysteines predicted to be used in disulfide bond formation. Arrows represent the catalytic site of LysA.


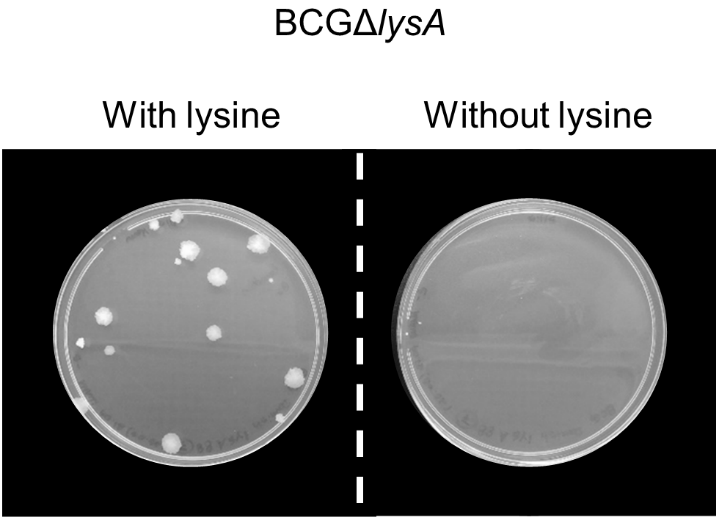


**Supplementary Figure 5. Serial passage of BCG*ΔlysA* does not reverse to the wild-type phenotype.** A strain of BCGΔ*lysA* with minimal deletion (deletion of 2 bp) was cultured for 8 passages in MB7H9-OADC supplemented with lysine. At the 8^th^ passage the culture was spread on plates with and without lysine. Inability to grow without lysine is the characteristic expected for auxotrophic organisms.


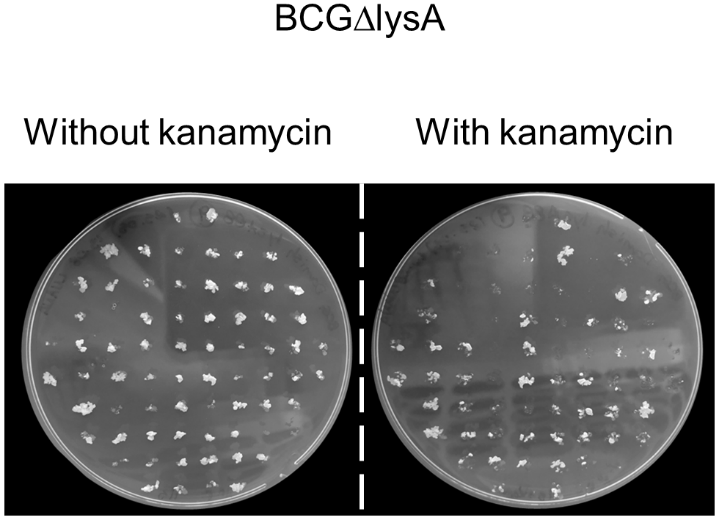


**Supplementary Figure 6. Curing auxotrophic clones of BCG transformed with pKLM-CRISPR-*lysA(x)*.** Following transformation of BCG with pKLM-CRISPR-*lysA(x)*, induction of Cas9 expression with tetracycline, and selection of auxotrophic BCG strains, 2 mirror plates were prepared with and without kanamycin. The colonies susceptible to kanamycin are those that have lost the plasmid and can be further used with the complementation vectors.


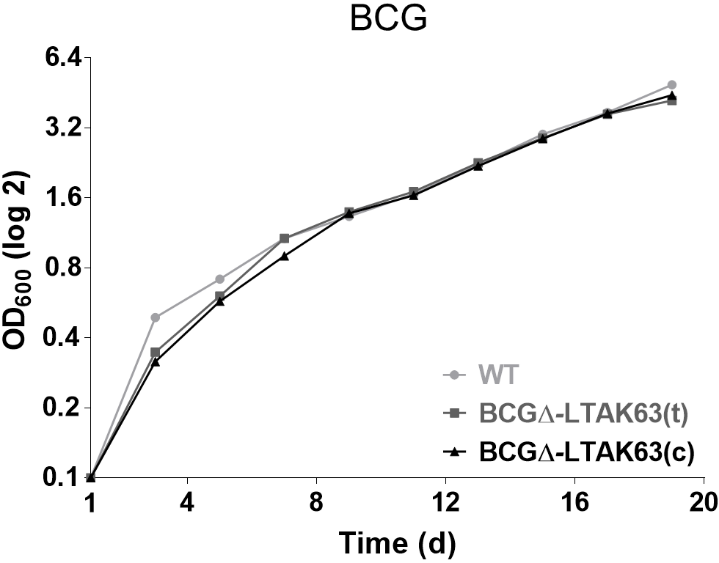


**Supplementary Figure 7. Growth curves of complemented auxotrophic strains are comparable to the wild-type strain.** After the generation of a functional LysA knockout, the strains were complemented with pAN71-*ltak63-lysA*(t) and pAN71-*ltak63-lysA*(c) generating BCGΔ-LTAK63(t) and BCGΔ-LTAK63(c), respectively. After an initial inoculum at OD_600_ 0.1, regular readings were measured on a spectrophotometer growth curves compared to the wild-type strain (WT).

**Supplementary Table 1. Characteristics of plasmids used and constructed in this study.**

| **Vector** | **Relevant Characteristics** | **Reference** |
| --- | --- | --- |
| pJH152 | Kan^R^ (Tn10), *E. coli* origin (pUC), of replication in mycobacterium origin (ori-myco), pα-Ag promoter, MSC, MSP-1, and the *lysA* gene. | (31) |
| pUC57 | *lacZ*, bla (Ap^R^), MCS, rep (pMB1)- 2710bp | GenScript |
| pUC57-cas9 | pUC57, carrying *cas9* codon optimized cassette | This study |
| pUC57-*tetR* | pUC57, carrying *tetR* cassette | This study |
| pUC57-*tracrRNA* | pUC57, carrying *tracrRNA* cassette | This study |
| pKLM-CRISPR-*lysA*(x) | pJH152, carrying *cas9* codon optimized, *tetR* and *tracrRNA* cassettes | This study |
| pLA71 | Mycobacterial promoter (BlaF*), Kan^R^ -12000 bp | (32) |
| pAN71-*ltak63* | pLA71, substituting PAN promoter for PBlaF*,  carrying the codon optimized *ltak63* | (5) |
| pAN71-*ltak63*-*lysA*(t) | pAN71-*ltak63*, carrying *lysA* gene in tandem and the deletion of Kan^R^ gene | This study |
| pAN71-*ltak63*-*lysA*(c) | pAN71-*ltak63*, carrying *lysA* gene in cassette and the deletion of Kan^R^ gene | This study |
| pCas | repA101(Ts) kan Pcas-*cas9* ParaB-Red *lacIq* Ptrc-*sgRNA*-pMB1 | (33) |

**Supplementary Table 2. Oligonucleotides used as primers in PCR reactions, in genome sequencing, or as the specific sequences of crRNA.**

| **Probe** | **Sequence 5’-3’** | **PCR product** |
| --- | --- | --- |
| crRNA-*lysA20*-BCG | GGGAGTGCAAACGTCCAGACAGAG/  AAACCTCTGTCTGGACGTTTGCAC | Specific sequence of sgRNAs to target *lysA* in BCG |
| crRNA-*lysA88*-BCG | GGGAGTCGGAAAGCACTGCGAAAG/  AAACCTTTCGCAGTGCTTTCCGAC | Specific sequence of sgRNAs to target *lysA* in BCG |
| crRNA-*lysA394*-Smeg(-) | GGGATGCACCGTGATCCGCGAGGCCGG/  AAACCCGGCCTCGCGGATCACGGTGCA | Specific sequence of sgRNAs to target *lysA* in *M. smegmatis at negative DNA strand* |
| crRNA-*lysA820*-Smeg | GGGACAGATGTCCATCGTGGATCTCGG/  AAACCCGAGATCCACGATGGACATCTG | Specific sequence of sgRNAs to target *lysA* in *M. smegmatis* |
| crRNA-*lysA123*-Smeg | GGGAGGTTCGCGGTGCCGACGGGGTGG/  AAACCCACCCCGTCGGCACCGCGAACC | Specific sequence of sgRNAs to target *lysA* in *M. smegmatis* |
| *LysA* | TAGCAGCTGGCGGCCGCTCATTGACCCTTCACCTCCAGG CTCAG/  TAGCAGCTGGCGGCCGCTCATTGACCCTTCACCTCCAGG CTCAG  TAGCAGCTGGCGGCCGCTCATTGACCCTTCACCTCCAG GCTCAG/  *TAG*CAGCTGGCGGCCGCTCATTGACCCTTCACCTCCAGGCTCAG | Sequencing of *lysA*  *lysA* amplification for cloning |
|  |  |  |
| Cas9 | TGGCATCCGTGGCGCGGCCGC AGAAATATTGGA/  TCGCTGGCATCGATAAAGGGGACCTCTAGGG | Sequencing of cas9 |
| TetR | TATCGATGCCAGCGAGTCATGAGGTNAC/  TCTACGTA GTCGACGCATGCCTCGAG | Sequencing of tetR |
| sgRNA | GCGTCGACTACGTAGAATTCAGAAATATTGGATCGTCGG/  CTAGTTAACTACGTCGACATTCTAGATTAATTAAAAAAAAGCACCGACTCGGT | Sequencing of sgRNA |
